# Optical Nano Antennas: State of the Art, Scope and Challenges as a Biosensor Along with Human Exposure to Nano-Toxicology

**DOI:** 10.3390/s150408787

**Published:** 2015-04-15

**Authors:** Abu Sulaiman Mohammad Zahid Kausar, Ahmed Wasif Reza, Tarik Abdul Latef, Mohammad Habib Ullah, Mohammad Ershadul Karim

**Affiliations:** 1 Department of Electrical Engineering, Faculty of Engineering, University of Malaya, 50603 Kuala Lumpur, Malaysia; E-Mails: zhdksr56@gmail.com (A.S.M.Z.K.); tariqlatef@um.edu.my (T.A.L.); 2 Faculty of Law, University of Malaya, 50603 Kuala Lumpur, Malaysia; E-Mail: ershad@siswa.um.edu.my

**Keywords:** biosensor, optical nano-antenna, nano-toxicology

## Abstract

The concept of optical antennas in physical optics is still evolving. Like the antennas used in the radio frequency (RF) regime, the aspiration of optical antennas is to localize the free propagating radiation energy, and *vice versa*. For this purpose, optical antennas utilize the distinctive properties of metal nanostructures, which are strong plasmonic coupling elements at the optical regime. The concept of optical antennas is being advanced technologically and they are projected to be substitute devices for detection in the millimeter, infrared, and visible regimes. At present, their potential benefits in light detection, which include polarization dependency, tunability, and quick response times have been successfully demonstrated. Optical antennas also can be seen as directionally responsive elements for point detectors. This review provides an overview of the historical background of the topic, along with the basic concepts and parameters of optical antennas. One of the major parts of this review covers the use of optical antennas in biosensing, presenting biosensing applications with a broad description using different types of data. We have also mentioned the basic challenges in the path of the universal use of optical biosensors, where we have also discussed some legal matters.

## 1. Introduction

The optical antennas, which represent unique optical detectors equivalent to radio frequency (RF) antennas, are a novel concept in the field of physical optics [1]. The optical antenna is an helping tool for influencing and regulating radiation in the optical regime. Nowadays, optical antennas are subjected to an increasing amount of technical research. This technology has potential in the enhancement of the efficiency of sensing, light emission, photo-detection, spectroscopy, and heat transfer [1]. Conventionally, optics and photonics are involved in the regulation of optical propagation using fibers, lenses, mirrors, and different diffractive components. In almost all areas, antennas are universal, covering satellite to toys. As optical antennas have numerous prospects, the key benefits of this type of antennas can be précised as follows:

Optical antennas:

(i) are point detectors which secure a recognition space of almost the square of the wavelength [2].
(ii) combine optical radiation into minute volumes for generating currents in the wire which are identified by a rectifying component of almost 0.02 µm^3^ volume. This minute material volume permits one to achieve faster responses. Initial assessments of this response time are about 100 ns for devices without optimization [3]. Conversely, one of the rectifying tools employed in detecting the signal is constructed on the basis of a tunnel effect, which has a response time of approximately 10–14 s, 10–15 s [4].
(iii) are known as polarization-sensitive sensors like the RF versions [2].
(iv) are capable of being tuned to a particular wavelength region. At optical frequencies, the metallic structures have a lossy character and as a result, the resonances are likely to be widened, which possibly limits the tuning ability [5].
(v) are directionally sensible subject to the metallic structure design and the addition of peripheral optical devices [6].

Though the optical antenna has use possibilities in numerous fields, it has a great possibility for use as a biosensor and this review only highlights the biosensing application. This review provides a clear overview of optical biosensors to the reader, a concept that arises from the contact of visible light with free electrons at a metal-dielectric boundary [7].

## 2. State of the Art

### 2.1. History of Optical Antennas

The root of the theory of optical antenna can be found in near-field optics [8]. The proposal of using a colloidal gold nanoparticle for optical radiation concentration on a model surface to overcome the restrictions of diffraction in imaging is first made by Synge in 1928 [9]. The concept of using gold nanoparticles as an antenna was first presented in 1985 by Wessel [10] and it was first demonstrated experimentally by using a gold-coated polystyrene particle by Fischer *et al.* in 1995 [11]. In the succeeding years, sharply pointed optical antennas were used in microscopy and spectroscopy [12,13,14]. Tip-enhanced near-field optical microscopy is the result of these experiments. In early 1968, optical antennas were utilized as whisker diodes in infrared radiation recognition and combination [15,16,17] and as a continuation of these studies, various investigations about infrared antenna structures have been done [18,19,20].

**Figure 1:**
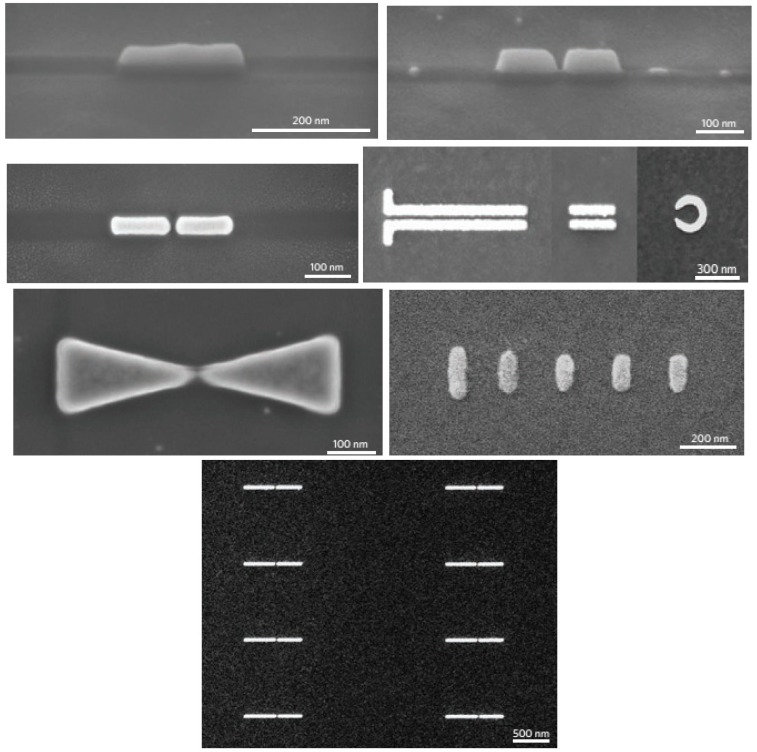
Optical antennas of different shapes.

In 1997, after proof of principle experiments, bow-tie type antennas have been suggested as optical probes for the near-field regime [21]. Later investigations presented the fabrication of bow-tie type antennas on tips [22]. After the establishment of the similarity of optical antennas with near-field optical probes [8], tip-on-aperture probe techniques become popular to grow the antenna structures [23,24]. As a result of these advances, many researchers head off to explore various antenna geometries with both experimental and theoretical approaches. As an example, Figure 1 displays several antenna shapes fabricated using different techniques. Nowadays, the use of surface plasmon resonance in optical antennas makes them more efficient for selected frequencies which holds potential for sensing and detection in the field of biology [18,25,26,27,28,29,30,31,32].

### 2.2. Physical Properties of Optical Antenna

Before entering in depth into the field of optical antennas, we should know their basics. The main parameters for designing optical antennas are:

#### 2.2.1. Local Density of Electromagnetic States (LDOS)

In the discussion of antennas, one of the most significant parameters is impedance. According to circuit theory, impedance is defined as *Z* = *V*/*I*, where *I* is current and *V* is voltage. According to this definition, the antenna is connected to the source through a transmission line, but this definition of antenna input impedance needs to be modified due to the feeding of optical antennas by confining light emitters rather than real currents. A practical replacement of this definition comprises the LDOS. This LDOS is the cause of the dipole energy dissipation in a random inconsistent environment. The allowance of a clear relationship of quantum-conventional formalisms is the main benefit of using the LDOS. LDOS is represented by ρ and the total LDOS can be found as [28]:

(1) ρ(r0,ω)=〈ρp(r0,ω)〉=2ωπc2Im{Tr[G↔(r0,r0,ω)]}

where *Tr* indicates the trace, ρ*_p_* is the partial LDOS, ω is the transition frequency, *G* is the Green function tensor, *c* is the velocity of light, and *r*_0_ is an arbitrary location. Therefore, the LDOS accounts for the existence of the antenna and is an extent of its properties. In the absence of an antenna in free space, we achieve ${\text{ρ}}_{p}={\text{ω}}^{2}/\left({\text{π}}^{2}c^{3}\right)$ and $\Gamma_{0}={\text{ω}}^{3}{\left|\langle g\left|\hat{p}\right|e\rangle \right|}^{2}/\left(3{\text{πε}}_{0}hc^{3}\right)$. Purcell observed the dependency of the amount of atomic decay on the indigenous atmosphere in 1946 [33]. Since then, it has been used for different systems, such as near interfaces of molecules [34] or atoms in cavities [35,36]. The adaptation of atomic decay rates has a foundation in the interface of the atom-distinct secondary field. This distinct field attains the rear of the position of the atom after scattering in the indigenous surroundings. The transition frequencies and energy states are also infected by this back-action [37,38].

#### 2.2.2. Antenna Impedance

According to circuit theory, the antenna resistance can be calculated as Re{*Z*} = *P*/*I*^2^. In an optical antenna, there is a governing dipole rather than a physical current which is more suitable for expressing *Z* according to the current density, j~iωp, as a replacement for the current, *I*. The antenna impedance, thus, can be defined as in [32] by the expression:

(2) Re{Z}=π12ε0ρp(r0,ω)

Therefore, the antenna resistance Re{*Z*} can be linked with the LDOS. The unit of antenna impedance is Ohm per area in place of the typical Ohm. Here, *Z* is mutually dependent on the position *r*_o_ and alignment *n_p_* of the dipole. According to Greffet *et al.* [32], the stored energy can be found by the imaginary part of *Z*.

#### 2.2.3. Antenna Efficiency

**Figure 2:**
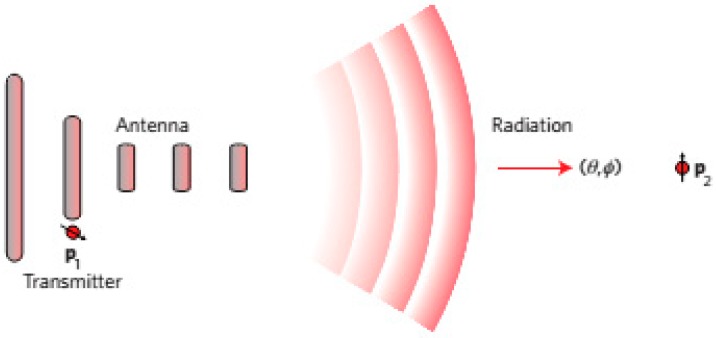
Enhancement of the transmission efficiency from the *Tx* to *Rx*.

A basic problem in antennas is demonstrated in Figure 2. This figure contains dipoles p1 and p2, which are represented as a transmitter (*Tx*) and receiver (*Rx*). Here, the function of the antenna is to boost the *Tx* to *Rx* transmission efficiency, which can be achieved by raising the *Tx* radiation, for which a suitable figure of merit is the antenna efficiency and this antenna efficiency can be found as in [1]:

(3) $${\text{ε}}_{rad}=\frac{P_{rad}}{P}=\frac{P_{rad}}{P_{rad}+P_{loss}}$$

where *P* is the total antenna dissipated power and *P_rad_* and *P_loss_* means radiated power and power loss, respectively.

#### 2.2.4. Directivity

The capacity of focusing the radiated power into a definite route is known as the directivity of the antenna, which represents the density of the angular power in relation to an isotropic radiator. The improvement of the efficiency of transmission can be accomplished by guiding the radiation towards *Rx*. Directivity is a measure of the proficiency for this system which can be represented as [1]:

(4) $$D(\theta ,\phi )=\frac{4\pi }{P_{rad}}p(\theta ,\phi )$$

where both θ and *ϕ* denote the direction of observation and *p*(θ, *ϕ*) denotes the angular density of power.

#### 2.2.5. Gain

Antenna gain is the result of the combination of antenna efficiency and directivity. The definition of antenna gain is similar to that of the directivity, but here the normalization is done in comparison with power *P* instead of the radiated power *P_rad_*. It can be mathematically represented as [1]:

(5) $$G=\varepsilon_{rad}D=\frac{4\pi }{P}p(\theta ,\phi )$$

Directivity and gain are generally calculated in decibels. As isotropic perfect radiators are impractical, a more realistic approach is to state an antenna of known configuration. Then the comparative gain can be demarcated as the fraction of the gain in a specified direction to the gain of a reference antenna in a similar direction [28]. Bouhelier *et al.* recently described the relative gain of optical antennas, using the dipole-like radiation from single nanoparticles as a reference [39,40].

#### 2.2.6. Reciprocity

Reciprocity makes it possible to trade off the sources and fields shown in Figure 2 to provide p_1_ · E_2_ = p_2_ · E_1_, where E_1_(E_2_) is the field of dipole p_1_(p_2_) calculated at the position of p_2_(p_1_). As a result, a noble transmitting antenna is also a noble receiving antenna. The reciprocity leads towards a correlation of emitter’s stimulation rate (*Γ_exc_*) with the impulsive discharge rate (*Γ_rad_*) for a two-state quantum emitter which can be presented as [28,41]:

(6) $$\frac{\Gamma_{exc,\theta }(\theta ,\phi )}{{\Gamma^{o}}_{exc,\theta }(\theta ,\phi )}=\frac{\Gamma_{rad}}{{\Gamma_{rad}}^{o}}\frac{D_{\theta }(\theta ,\phi )}{D_{\theta }^{o}(\theta ,\phi )}$$

where the meaning of the superscript “*o*” is the nonexistence of the antenna, while the subscript “θ” specifies the nature of polarization; explicitly, the points of the electric field in the direction of unit vector θ. An alike equation can be formed for polarization in the direction of *ϕ*. It is interesting that the excitation in high directivity direction allows *Γ_exc_* to be boosted more intensely than *Γ_rad_*. From Equation (6), it is clear that the relation of *Γ_exc_* and *Γ_rad_* due to the existence of the antenna is proportionate and has been included qualitatively in several studies [22,41,42,43].

#### 2.2.7. Antenna Aperture

Antenna aperture is another significant antenna parameter, which is similar to the absorption cross-section σ. Let, a dipole with σ*_o_* cross-section as an *Rx* and the *Rx* is not connected to an antenna, *n_p_* is a directional unit vector, and *E_o_* is the receiver incident field. The receiver field increases to *E* after its connection with the antenna and antenna aperture (or absorption cross-section) can be found as

(7) $$\text{σ}=\frac{{\text{σ}}_{o}{\left|n_{P}\cdot E\right|}^{2}}{{\left|n_{P}\cdot E_{o}\right|}^{2}}$$

Therefore, the antenna aperture changes with the indigenous intensity improvement factor. Many studies have revealed that 10^4^–10^6^ intensity enhancements are possibly attainable [44,45,46] and therefore, a layer of molecules (all molecules are attached to an optical antenna) situated 0.1–1 μm apart are capable of absorbing all of the incident radiation for distinctive molecules with 1 nm^2^ free-space cross-sections. Obviously, this evaluation has a limited validity because it overlooks the coupling among the antennas.

#### 2.2.8. Effective Wavelength

In case of the radio wave antennas, the radio frequency (RF) counterpart of optical antennas, the wavelength of the incident radiation, λ relates to its design rules. For instance, the length of a half-wave antenna *L* is λ/2, and the separation between elements of a Yagi-Uda antenna corresponds to some fraction of λ [47,48]. Therefore, the scaling of RF antenna design from one wavelength to another is very straightforward because of its proportionality to λ. Conversely, this scaling is not applicable to the optical regime where the diffusion of emission into metals has to be considered. The delay after supplying the driving field to get the electronic response due to a finite electron density results in the skin depth and this skin depth is usually bigger than the antenna element diameter. Hence, the electrons of a metal react to an effective wavelength λ*_eff_* instead of the wavelength λ. This effective wavelength can be determined as [49,50]:

(8) $${\text{λ}}_{eff}=n_{1}+n_{2}\left(\frac{\text{λ}}{{\text{λ}}_{P}}\right)$$

where *n*_1_ and *n*_2_ are geometric constants and λ*_p_* is the wavelength of the plasma. Applying the new wavelength calculation rule, the wavelength of a half wave antenna for an optical regime becomes λ*_eff_*/2 instead of λ/2. The difference between λ and λ*_eff_* is influenced by the geometric constants, but is normally between 2 to 5.

#### 2.2.9. Conductivity of Antenna Materials

As the conductivity of metals drops considerably when the diameters become less than 5 nm, metals are possibly not the best selection for antenna elements. In the case of diameters of less than 5 nm, carbon nanotubes are superior conductors than metals [51]. Therefore, on a small scale, carbon materials have wide possibilities to be the materials of choice for optical antenna elements [52].

#### 2.2.10. Antenna Resonance

The resonantly excited nanostructures behave as optical antennas similar to RF antennas, especially in IR (infrared) that concentrate the energy of electromagnetic radiation to a confined volume of the sub-wavelength scale. Thus, nanorods, with µm-sized lengths *L* that show plasmonic resonances in the IR spectral range [53,54,55] are termed nanoantennas. However, the simple λ/2-dipole behavior known from RF antennas, where the relationship between *L* and the resonant wavelength λ*_res_* is given by 2*L* = λ*_res_*, does not hold for nanoantennas at optical frequencies. Moreover, the finite penetration depth of the light into the metal, and the non-negligible diameter *D* of the antenna lead to the modified relation [53].

(9) $$2L=c_{2}\left[\frac{{\text{λ}}_{res}}{{\text{λ}}_{p}}\right]-c_{1}$$

In this equation, λ*p* denotes the plasma wavelength of the antenna’s material, whereas the coefficients *c_1_* and *c_2_* rely on *D* and on the static dielectric constant ε*_s_* of the surrounding medium. The basic assumption in this model is a high aspect ratio of the antenna (*D* << *L*) and the metal is described as a free-electron gas, as stated by the Drude model with negligible relaxation rate compared to photon frequencies. Since these conditions are adequately satisfied for gold nanorods with *L*/*D* > 10 in the mid-IR region, Equation (9) can delineate the resonance behavior of isolated or at least non-interacting nanoantennas.

### 2.3. Challenges and Outlook of Optical Antennas

There are a number of challenges to the broad use of optical antennas and they must be addressed before the technology becomes extensively usable.

#### 2.3.1. Following the Analogy of RF Antenna

**Figure 3:**
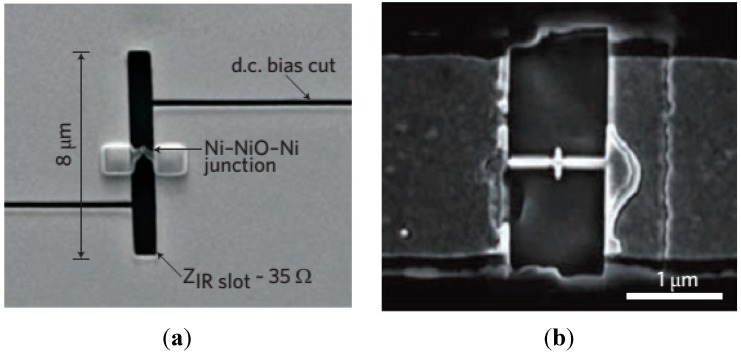
SEM images of (**a**) Slot antenna (**b**) Open-sleeve dipole antenna.

How far has the similarity between optical and RF antennas been achieved? Different nanofabrication tools have been extensively used for achieving such similarity. However, the RF antennas are driven locally at the feed gap, while the far-field was used to drive the initial optical antennas. For detection in the infrared regime, antenna-mediated transduction has been investigated, for instance, a slot antenna [56] or a dipole antenna (Figure 3) [57]. Conversely, a huge number of enhanced RF antennas are needed to investigate in the optical regime. On behalf of directional recognition, customary loop and travelling-wave antennas are evident candidates. Specifically, the Yagi-Uda antenna scheme was practiced by a few researchers [58,59,60], who decided that the radiation at the feed component of an optical source can be totally focused in a single cone. Recently, experimental demonstration was made for the directional scattering from an optical Yagi-Uda antenna array [47,48].

Present RF antennas are greatly improved and enhanced in terms of dimension and bandwidth. The idea of using the fractal design of cellular phones in optics was first introduced by Stockmann [61]. Following this idea, modern fractal antennas can be designed for capturing and channeling a wide band of light to a single point, for instance the Sierpinski carpet [62]. In reality, the smallest dimension is a challenge, which sets a limitation on the operational frequency.

The directionality of the antenna can be controlled by impedance match regulation or by the use of a phased array, which permits quick routing of beams. An optical phase antenna has been suggested in [63] for attaining near-field focusing, but this type of phase controlling for a coupled optical antenna is still a challenge.

#### 2.3.2. Impedance Matching

Impedance matching between source and antenna is one of the main challenges in designing an optical antenna. For a molecule-like source, approximately 1 MΩ reactive impedance can be acquired by considering the source as a capacitor, similar to a plate of dimension 0.2 nm × 0.2 nm with a similar separation [1]. Conversely, a typical metal nanostructure has an impedance commonly in Ohmic range and is exceptionally small, for instance ~3 Ω impedance is found for a half-wave linear antenna [1,28]. At present, the reimbursement process for this huge impedance gap is indistinct, but impedance matching is vital for effective connection between the source and radiation field. The initial platforms have been established [60,61,64,65], and for a half-wave dipole antenna, it has been presented that adjusting the feed-gap impedance allows proper tuning of the antenna resonance [66,67,68].

An atom’s impedance cannot be fully imaginary due to the impulsive emission. The radiation losses must be explained by the real part of the impedance. For effective radiation, the excited state lifespan of an atom must be trivial. Therefore, the radiation resistance must be connected to LDOS [28,69]. Coupling to an optical antenna raises the LDOS, which makes the source (atom/molecule) a more proficient emitter [43,44,70,71].

#### 2.3.3. Electro-Optical Conversion

In both of the RF and microwave frequencies, the main use of antennas is to transform electric currents into electromagnetic radiation, and *vice versa*. Conversely, the operational base of most optical antennas is “light-in, light-out”. A small number of studies have reported the conversion of optical radiation into photocurrents by using antennae [57,72]. This transduction can be inspired by different high-frequency devices, like whisker diodes for infrared [15,17] and from photon radiation in scanning tunneling microscopy [73,74]. This type of infrared whisker diodes are able to deliver a pathway to *Rx* antennas [18]. The main difficulty in infrared whisker diodes is the metal-oxide-metal junction’s capacitance-dependent high frequency cut off [56]. In contrast, scanning tunneling microscopy can decay radiatively according to the stimulation of surface plasmons by electrons [75,76]. However, evolving nanofabrication technology makes it promising to work with much reduced lengths, and it is anticipated that this type of conversion (electro-optical) by using the coupling of electron and plasmon will be achieved at some point.

#### 2.3.4. Selection Rules

Here, we have already indicated little significant dissimilarity between optical antennas and RF antennas. For instance, the penetration of radiation into metals at optical frequencies cannot be ignored, and as a result, optical antennas react to the effective wavelength instead of wavelength of the inward radiation. Additionally, metals do not behave linearly in the optical regime, which creates the possibility of mixing and converting dissimilar frequencies. Lastly, but most significantly, the local fields near the optical antenna have spatial magnitudes that come near the length scale of the molecular quantum wave functions. This forms communication channels that are illicit by the selection rules of typical electric dipoles [28]. Moreover, the solid localized field near an optical antenna increases the momenta of photons up to the order of electron momenta in matter, and therefore increases the conventionally momentum-illicit alterations. Though interactions in the near-field beyond the rules of dipole selection are subject to theoretical studies [77], experimental verification is still needed.

#### 2.3.5. Reproducibility and Repeatability

**Figure 4:**
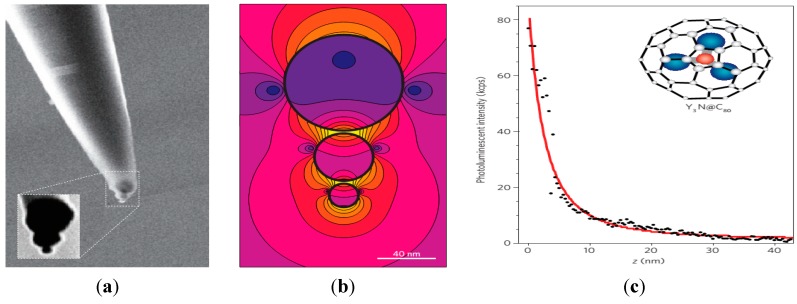
Sample of optical antenna using bottom-up fabrication. (**a**) SEM image of a gold trimer antenna; (**b**) intensity nearby a trimer antenna at 650 nm wavelength; (**c**) Fluorescence from a metallofullerene [81].

The progress of an optical antenna application depends on the capability of fabricating it with adequate material properties and precision on a nano-scale. Usually, the bottom-up technique has delivered good-quality crystalline metallic nanoparticles of manageable form and dimensions of a few nanometers. This type of colloidal nanoparticles display high-grade resonances (*Q* > 10) overcoming the restrictions of the dielectric properties of metals [78]. Additionally, triangle-, star-, core-shell-, and pentagon-like particles have been manufactured [79,80]. Although the fabrication (Figure 4) of antenna probes has been accomplished by selecting distinct or manifold gold nanoparticles, the composition of a designed antenna geometry by arranging the colloidal particles is still a challenge [70,71,81,82]. All of the replication is fundamentally dependent upon the skill and serenity of the researcher. Nowadays, prolonged optical antenna arrays of Au or Ag nanoparticles are usually fabricated through electron-beam lithography techniques [83]. Though 10–20 nm accuracy was achieved (Figure 4), the metals usually remain polycrystalline with 10–30 nm grains, which usually results in low quality (*Q* ~ 5) resonance, and thus, just one or two grains can affect the properties of specific antennas.

For more complex 3-D structures, focused ion beam milling is an suitable substitute for electron-beam lithography [33]. Moreover, reproducibility is a matter of patience. Though antenna nanofabrication methods have advanced swiftly, the foremost difficulty at the present is how to provide for coupling between the antennas and the active optical sources, like nitrogen-vacancy centers, molecules, and quantum dots. For identifying the hot spots, post-selection can be used and thus the enhancement of antenna magnitude became pragmatic [44], where actual molecular antenna structures remained unidentified. Conversely, pre-patterned models are used in colloidal antennas [72]. In this case, more straightforward control has been attained by scanning antenna probes. Here, both of the comparative location and alignment are fully controlled, which gives an autonomous perception on the excitation improvement [41,70,71]. The perfect direct nanopositioning of particular quantum emitters in conjunction with complete control of the position and alignment corresponding to the antenna are the real test for future practical applications. Based on these physical properties, researchers have made many applications of optical antennas for different fields, such as nanoscale imaging and spectroscopy, photovoltaics, light emission, coherent control, *etc.* Here, in this review, we only discuss optical antennas in the field of biosensors.

## 3. Optical Antennas as Biosensors

For twenty years, broad research has been done in the field of biosensors for their substantial uses, such as in detection of DNA, different types of cancer and biomolecules. Therefore, an evolving technology in present biosensor research is optical antennas for label-free and real-time molecular recognition. Nowadays, numerous researchers are studying making bio-sensing optical antennas. As optical antennas are governed by the rule of plasmonics, these studies are divided into two different types, according to the surface plasmons involved: (i) surface plasmon resonance (SPR) biosensors [84] and (ii) localized surface plasmon resonance (LSPR) biosensors [85].

**Figure 5:**
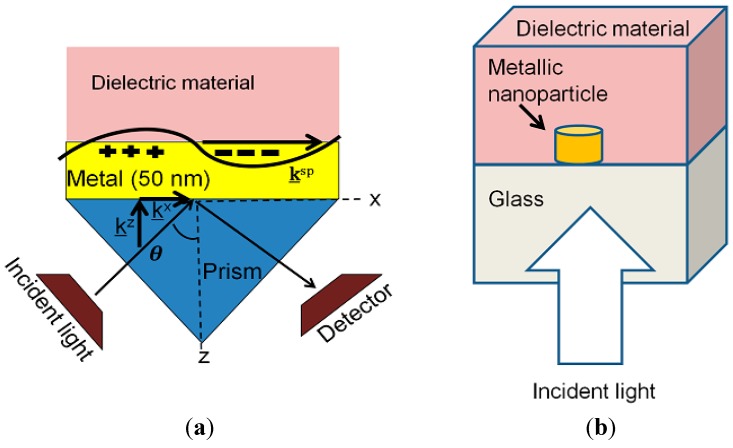
Demonstration of the principle of (**a**) SPR and (**b**) LSPR system.

Surface plasmon polaritons (SPP) are the basis of SPR biosensors. SPR is a charge-density oscillation of SPP that might present at the interface of two media with dielectric constants of opposite signs, for example, a metal and a dielectric. The charge density wave is associated with an electromagnetic wave, the field vectors of which attain their maxima at the interface and decay evanescently into both media. The simplest and low cost SPR configuration is the Kretschmann configuration (Figure 5a). Although SPR biosensors offer very high sensitivity, they are not adequate for small biomolecules in low concentrations. Recently, a new modulation technique has been employed, which surpasses the sensitivity of the standard SPR sensor. Therefore, these types of biosensors must be improved for practical use.

LSPR biosensors are based on localized surface plasmon (LSP) phenomena [85,86]. At the plasmon resonant frequency, the optical extinction of a nanoparticle become maximum, which is dependent on the adjacent medium’s refractive index and the nanoparticle’s size and shape. Variations in the refractive index of the adjacent medium are noticed by the changes in peak wavelength of the LSPR. This peak wavelength change is found by measuring the spectral extinction, as displayed in Figure 5b. The bigger nanoparticles are more sensitive [87], but their peaks are stretched due to radiative damping and multipolar excitations.

### 3.1. SPR Biosensors and Present Research

SPR biosensors are able to detect biomolecular interactions directly without any labeling and thus allow real-time assessment of the kinetics and concentrations of analytes, and their thermodynamic binding parameters. Different features of familiar SPR sensors have been listed in Table 1.

**Table 1:** Familiar SPR sensors and their performances.

Structure	Characteristics	Wavelength	Sensitivity	Reference
**Kretschmann configuration**				
**Typical sensor**	Au, Ag metal film	400–800 nm	100–300 deg./RIU	[88]
	Ag film, low index prism	1310 nm	500 deg./RIU	[89]
	Au metal film	500–1000 nm	7500 nm/RIU, 10^−8^ RIU	[90]
**Over layer**	Au and Si, ZrO_2_ thin film	632.8 nm	50–230 deg./RIU	[91]
	Ag-Au bimetallic layer	632.8 nm	7.85 × 10^−6^ RIU	[92]
**Nanostructured sensor**	Au nano cylindrical array	632 nm	10^−7^/RIU	[93]
	Au nanorod metamaterial	1200–1300 nm	30,000 nm/RIU	[94]
	M-D mixed grating	633 nm	~120 deg./RIU	[95]
**Multichannel sensor**	Dual channel, D over-layer	550–1150 nm	5 µg/mL α-DNA	[96]
	Angled polishing prism	500–900 nm	2710, 8500 nm/RIU	[97]
**Fiber-Optic SPR sensors**				
**Symmetrical cladding off**	Au, Ag metal film	400–650 nm	2000–4500 nm/RIU	[98]
**Grating**	Cascaded LPG	~1520 nm	−23.45 nm/RIU	
**Nano-structured sensor**	Au metallic grating	900–1600 nm	4000–9800 nm/RIU	[98]
**Nano-structured-coupling**				
**Grating coupling**	Au surface grating	~600 nm	440 nm/RIU	
	Al-Au bimetallic layer	900 nm	187.2 deg./RIU	[99]
**Metamaterial-like**	Au nano-structured layer	~150 THz (~2000 nm)	588 nm/RIU	[100]
**Nanoparticle based sensors**				
**Nano-structure**	Single or double-square periodic nanoparticle array	400–950 nm	200–350 nm/RIU	[101]
	Nanoparticle pair, disk pair	500–900 nm	172,434 nm/RIU	[102]
	Unperiodic array	300–700 nm	165 nm/RIU	[103]
	Gold nano-ring array	300–1800 nm	637.3 nm/RIU	[104]
**EOT based sensors**				
	Square nanohole array	600–1000 nm	300 nm/RIU	[88]
	Nanohole array	1520–1570 nm	1110, 1570 nm/RIU	[105]
	Fluoropolymer Substrates	~600 nm	323 nm/RIU	[106]
**Interferometer**				
	Mach-Zehnder type	~1550 nm	250 nm/RIU	[107]
	Two slit interference	877.3 nm	4547 nm/RIU	[108]
**Ring resonator**				
	Disk resonator	~1460 nm	600 nm/RIU	
	Triangular resonator	~1555 nm		[109]

Now, we are going to present different applications of SPR biosensors and their current research status.

#### 3.1.1. Insecticide Detection

The U.S. Environmental Protection Agency (EPA) establishes the higher limits of acceptable concentrations for most pesticides; for example, the limit of concentration for atrazine is 3 ng/mL and for simazine it is 4 ng/mL [110]. On the other hand, the European Union permits up to 0.1 ng/mL and 0.5 ng/mL, for those two insecticides, respectively. A list of uses of SPR biosensors for pesticide detection is provided in Table 2.

**Table 2:** SPR biosensors in pesticide detection.

Pesticide	Detection Range	Instrument	Reference
Atrazine	0.05–1 ng/mL	Biacore	[111]
Simazine	0.2 µg/L in	Integrated SPR	[112]
Atrazine	5 ng/mL in	Biacore 2000	[113]
Atrazine	1 ng/L–1 mg/L	Biacore 2000	[114]
2,4-Dichlorophenol	10–250 ppb	SPR-20	[115]
2,4-Dichlorophenoxyacetic acid (2,4-D)	0.5 ng/mL–1 µg/mL	SPR-20	[116]
Paraoxon	1–100 ppb	L-SPR	[117]
Chlorpyrifos	45–65 ng/L	β-SPR	[118]
Carbaryl	1.38 µg/L	β-SPR	[119]

#### 3.1.2. Explosive Detection

Nowadays, there is a vast demand for optical sensors for detecting different explosives. In Table 3, a list of SPR biosensors for explosive detection is provided.

**Table 3:** SPR biosensors for explosive detection.

Explosive	Detection Range	Instrument	Reference
Trinitrotolene (TNT)	8 ppt–30 ppb	SPR 670 M	[120]
2,4,6-Trinitrophenol (TNP)	10 ppt–100 ppb	SPR-760	[121]
TNT	0.09–1000 ng/mL	SPR-760	[122]
TNT	1 µg/mL	Spreeta	[123]
TNP	0.1 ppb	SPR-760	[124]
TNT	95 ppt	SPR-760	[125]
TNT	1–10 pg/µL	SPR, QCM	[126]
TNT	0.008–30 ng/mL	SPR-760	[120]

#### 3.1.3. Environmental Pollutant Detection

Today’s swift industrial development demands the pollutant detection for all livable atmospheres. Some SPR biosensor-based pollutant detectors along with their range is given in Table 4.

**Table 4:** SPR biosensor-based pollutant detection.

Pollutant	Detecting Range	Instrument	Reference
2,3,7,8-Tetrachlorodibenzo-*p*-dioxin	0.1 ng/mL	Biacore 2000	[113]
4-Nonylphenol	2 ng/mL	Biacore Q	[127]
Phenol, hydroquinone, resorcinol, phloroglucinol, and catechol	100 µg/mL	Intensity modulation SPR	[128]
Phenol	5 µg	Multiscope SPR	[129]
Bisphenol A (BPA)	10 ppb	SPR-20	[115]
3,3',4,4',5-pentachlorobiphenyl (PCB)	2.5 ng/mL	Biacore 2000	[113]
2-Hydroxybiphenyl (HBP)	0.1 ng/mL	Miniaturized SPR biosensor, 1-1,	[130]
Cadmium, zinc, and nickel	0.1 ng/mL in buffer	Biacore SPR	[131]
Copper ions (Cu^++^)	0.063 pg/mL–6.3 µg/mL		[132]

#### 3.1.4. Bacteria Detection

Normally, bacteria detection using SPR biosensors is challenging for several causes [133,134,135]. One of these challenges is the smaller penetration depth of a 100 nm SPR biosensor in comparison with the usual bacterium size of 1–5 µm. As a result, most of the cell binding takes place in the exterior of the transitory field. An additional cause is a smaller refractive index difference of the detection environments, such as the bacterial cytoplasm and aqueous environments. Finally, the inadequate amount of diffusion of bacteria in the direction of the biorecognition elements on the surface of the sensor. In spite of these challenges, a number of researchers have been working in this field. Table 5 shows information on SPR biosensors for detecting harmful bacteria.

**Table 5:** Detection of bacteria with SPR biosensors.

Bacteria	Used Assay	Range of Detection	Instrument	References
*Escherichia coli* O157:H7	Inhibition immunoassay	0.1–1 × 10^7^ cfu/mL	Biacore	[136]
*Escherichia coli* O157:H7		10^4^ cfu/mL	Multiscope SPR	[137]
*Escherichia coli* O157:H7	Direct immunoassay	8.7 × 10^6^ cfu/mL	Spreeta	[138]
*Escherichia coli* O157:H7		10^6^ cfu/mL in buffer 10^8^ cfu/mL in apple juice	Reichert SR7000 SPR	[139,140]
*Escherichia coli* O157:H7		10^5^–10^7^ cfu/mL	Custom made	[141,142]
*Salmonella typhimurium*		10^2^–10^9^ cfu/mL	Multiskop	[143]
*Salmonella paratyphi*		10^2^–10^7^ cfu/mL	Multiskop	[144]
*Salmonella enteritidis, Listeria monocytogenes*		10^6^ cfu/mL	Custom made	[145]
*Listeria monocytogenes*		10^7^ cfu/mL	Biacore 3000 SPR	[146]
*Bacillus subtilus spores*		10^7^ spores/mL	Scattering SPR	[133]
*Staphylococcus aureus*		10^7^ cfu/mL	Reichert SR7000 SPR	[147]
*Vibrio cholerae* O l		3.7×10^5^ cfu/mL	Multiskop	[148]
*Legionella pneumophila*		10^5^ cfu/mL	Custom made	[149]
*Yersinia enterocolitica*		10^2^–10^7^ cfu/mL	Custom made	[150]
*Campylobacter jejuni*		1.1 × 10^5^ cfu/mL	Custom made	[141]
*Legionella pneumophila*		10^5^ cfu/mL	Custom made	[149]
*E. coli* O157:H7		10^5^ cfu/mL	Multi-channel SPR	[150]
*Staphylococcus aureus*		10^4^ cfu/mL	Spreeta	[151]
*E. coli* O157:H7	Sandwich immunoassay	1.4 × 10^4^ cfu/mL	Custom made	[141]
*S. choleraesuis*		4.4 × 10^4^ cfu/mL		
*L. monocytogenes*		3.5 × 10^3^ cfu/mL		
*C. jejuni*		1.1 × 10^5^ cfu/mL		
*Escherichia coli* O157:H7		5–7 × 10^7^ cfu/mL	Biacore	[151]
*Salmonella strains* from groups A, B, D, and E		1.7 × 10^3^ cfu/mL	Biacore	[152]
*Staphylococcus aureus*		10^5^ cfu/mL	Reichert SR7000 SPR	[147]
*Listeria monocytogenes*	Subtractive inhibition immunoassay	10^5^ cfu/mL in less than 30 min	Biacore 3000	[153]
*Listeria monocytogenes*		10^5^ cfu/mL	Biacore 3000	[154]

#### 3.1.5. Virus Detection

Numerous researches have been conducted in virus detection using SPR biosensors. Table 6 provides some examples with details.

**Table 6:** SPR biosensors for virus detection.

Detected Virus	Used Assay	Detection Limit	Instrument	Reference
Epstein-Barr virus	Direct immunoassay	0.2 ng/mL	Custom-made	[155]
Hepatitis B	Direct immunoassay	9.2 nm	Spreeta	[156]
	Sandwich immunoassay	4.39 nm		
	Peroxidase–anti-peroxidase complex	0.62 nm		
Cowpea mosaic virus	Direct immunoassay		Biacore	[157]
Human immunodeficiency virus type 1 (HIV 1)	Using specific hybridization of immobilized biotinylated HIV-1		Biacore 1000	[158]

#### 3.1.6. Toxin Detection

Though most toxins can be detected instantly at higher density, normally for lower concentration, the sandwich assay is used. The recognition of bacterial toxins using SPR biosensors mainly relies on toxins’ molecular weight. Table 7 provides information about some SPR biosensors for toxin detection.

**Table 7:** Detection of toxins by SPR biosensors.

Toxin	Assay	Matrix	Detection Range	Instrument	Reference
Staphylococcal enterotoxin B (SEB)	Direct Immunoassay	Buffer	5 ng/mL	Wavelength Modulation SPR	[159]
	Sandwich Immunoassay	Buffer and milk	0.5 ng/mL		
SEB	Sandwich immunoassay	Buffer	2.5–50 ng/mL	Biacore 1000	[160]
SEB	Competitive immunoassay	Buffer	0.78–50 ng/mL	Biacore 1000	[161]
		Whole and skimmed milk	0.312–25 ng/mL		
SEB	Direct immunoassay	Buffer	In ng range	Fiber-optic SPR	[162]
SEB	Direct assay	Buffer	In nm range	Spreeta	[163]
	Sandwich immunoassay		In fm range		
Aflatoxins B_1_	Inhibition immunoassay	Buffer	3–100 ng/mL	Biacore 1000	[164]
Fumonisin B_1_ (FB_1_)	Direct immunoassay	Buffer	50 ng/mL	Custom-built SPR	[165]
Deoxynivalenol	Inhibition immunoassay	Naturally contaminated wheat samples in buffer	2.5–30 ng/mL	Biacore Q	[166]
Domoic acid	Inhibition immunoassay	Buffer	0.1–1000 ng/mL	Custom-built	[167]
	Competitive immunoassay		3 ppb (10 nM), from 4–60 ppb (13–200 nM)	Six-channel SPR, Spreeta	[168]
	Competitive immunoassay	Buffer	2 ng/mL to 3.3 µg/mL	Biacore 3000	[169]
Tetanus	Direct immunoassay	Buffer	0.028 Lf/mL	Wavelength modulation SPR	[170]

#### 3.1.7. Allergen Detection

Nowadays, the increased consciousness of allergens has led to rising calls for consistent, fast, and sensitive locating and measuring devices for allergens. Use of SPR biosensors for allergen detection is shown in Table 8.

**Table 8:** Detection of allergen by SPR biosensors.

Allergen	Detection Limit	Instrument	Reference
Peanut proteins	0.7 µg/mL	Miniaturized SPR Biosensor	[171]
Soy, pea, and soluble wheat proteins allergens	Below 0.1% of plant protein in the total milk protein content	Biacore 3000	[172]
*β*-Casein	85 ng/mL	Biacore 3000	[173]
Histamine (3-imidazole)	3 ppb	SPR 20	[174]
Pollen of perennial rye grass		Biacore	[175]

#### 3.1.8. Biomedical Analyte Detection

Many SPR biosensors have been used in the field of biomedical analyte detection. Table 9 presents a list of these.

**Table 9:** SPR biosensors for biomedical-analyte detection.

Analyte	Matrix	Assay	Detection Range	Instrument	Reference
***Myocardial-damage markers***											
Myoglubin and Tropinon I	Buffer	Direct immunoassay	3 ng/mL	Two-channel multi-mode SPR	[176]
Tropinin T		Direct immunoassay	0.01 ng/mL	SPR	[177]
Human Tropinone I		Direct assay	2.5–40 ng/mL	Wavelength modulation SPR	[178]
		Sandwich immunoassay	0.5-20 ng/mL		
Fatty acid binding protein (H-FABP)	Buffer	Competitive immunoassay	200 ng/mL	Planar SPR and fiber optic-SPR	[179]
***Cancer markers***											
Prostate-Specific Antigen (PSA)	Buffer	Direct assay	0.15 ng/mL	IBIS II dual channel SPR	[180]
		Sandwich immunoassays	2.4 ng/mL		
	Serum	Direct enhancement	10 ng/mL	Biacore 2000	[181]
Interleukin-8 (IL-8)	Buffer	Sandwich immunoassay	2.5 pM	Biacore	[182]
	Saliva		184 pM		
***Hormones***					
Estrone and estradiol	Buffer	Inhibition immunoassay	0.01–3000 ng/mL	Biacore	[183]
17-β-Estradiol	Buffer	Inhibition immunoassay	0.47–21.4 nM		[184]
Progesterone	Buffer	Indirect inhibition immunoassay	143 pg/mL	Biacore	[185]
Insulin growth factor-1	Milk	Direct immunoassay	0.5–1 ng/mL	Biacore	[186]
Human chronic gonadotropin (hCG)	Buffer		0.05–1 µg/mL		[187]
***Drugs***					
Morphine	Buffer	Inhibition immunoassay	0.1–10 ng/mL	SPR and QCM	[188]
Morphine-3 glucronide (M3G)	Buffer	Indirect inhibition immunoassay	0.7 ng/mL	Biacore 1000	[189]
	Dilute urine		2.4 ng/mL		
Anti-thrombotic agent Fragmin	Buffer	Inhibition immunoassay	625 ng/mL	Biacore 3000	[190]
7-Hydroxycoumarin	Diluted serum	Competitive and Inhibition immunoassays	0.5–80 µg/mL	Biacore	[191]
Oral anticoagulant 4'-aminowarafrin	Plasma samples	Inhibition immunoassay	4–250 ng/mL	Biacore 3000	[192]
β-Lactam antibiotics	Milk	Direct inhibition	4 µg/kg	Biacore 3000	[193]
β-Lactam antibiotics (penicillin G)	Milk	Inhibition immunoassay	1.2 µg/kg	Biacore Q	[194]

### 3.2. LSPR Biosensors and Associated Surface Enhanced Phenomena

A number of research groups are now trying to find alternate approaches for optical bio-sensing using the surprising optical characteristics of different nanoparticles. Nanoscale biosensors can be achieved with the shifts in LSPR [195,196,197]. Generally, LSPR biosensors work in a similar way as SPR sensors by transferring small refractive index variations into an assessable wavelength shift as follows [198,199]:

(10) $$\Delta {\text{λ}}_{\max}=m\left(n_{sample}-b_{blank}\right)\left[1-\exp\left(-\frac{2d_{sample}}{l_{d}}\right)\right]$$

Here, *m* is the sensor’s refractive-index sensitivity, $n_{blank}$ and $n_{sample}$ are the refractive indexes of the bulk and sample environment, correspondingly, $d_{sample}$ is sample layer’s effective thickness, and *l_d_* is the decay length of the characteristic electromagnetic field related to the sensor. Some differences between SPR and LSPR sensors are shown in Table 10.

**Table 10:** Differences between SPR and LSPR sensors [96,110,199].

Different Parameter	SPR	LSPR
Refractive index sensitivity	~2 × 10^6^ nm/RIU	~2 × 10^2^ nm/RIU
Overall sensitivity	The sensitivity of LSPR sensors are better than that of the traditional SPR sensors without metallic nanostructures	
Decay length	~200 nm	~6 nm
Throughput	LSPR technology has high-throughput screening capabilities in a highly compact design	
Controls over angle of incidence	Needs precise control	No precise control is needed
Controls over ambient temperature	Needs precise control	No precise control is needed
Nature of Measurement	Invasive	Non-invasive
Use in *in vivo* quantification	LSPR is better for *in vivo* quantification than SPR	

Other optical phenomena related to LSPR are also enhanced, for example surface-enhanced Raman scattering (SERS) and surface-enhanced fluorescence (SEF), as a result of the indigenous electromagnetic field enhancement nearby the nanoparticle [200]. Some applications of LSPR biosensors and associated surface phenomena are given below.

#### 3.2.1. Wavelength-Shift Based Application

The responsiveness of LSPR sensors in a dielectric atmosphere is very high, which is beneficial for the exposure of conformational variations and molecular binding events, and are able to provide both kinetic and steady-state information. The wavelength-shift of LSPR has been employed as a conversion scheme for investigating the binding interactions of molecules. Table 11 shows the sensitivities of different LSPR biosensors in the nanostructure range.

**Table 11:** Sensitivity of different LSPR sensors for wavelength shift-based recognition.

Structure	Dimension (nm)	λ_LSPR_ (nm)	Sensitivity (nm/RIU)	FOM ^1^ (RIU^−1^)	References
***Gold nanoparticles***					
Nanospheres	15	520	44	0.6	[197,201, 202]
	50		60		
	30		71		
	13		76		
Nanobranches	80	1141	703	0.8	[202]
Nanoshells hollow	50	680	409		[201]
Nanoshells/SiO_2_ core	50–175	Varies	570–996		[203]
Nanorings	75–150	1058–1545	880		[204]
Nanorods	74 (*d* = 33)	700	252		[202,205]
	40	653	195	2.6	
	55	728	224	2.1	
	74 (*d* = 17)	846	288	1.7	
Nanorice core	9.8–27.5	1160	800		[206]
Nanocubes	44	538	83	1.5	[202]
Nanobipyramids	27–189	645	15	1.7	[202]
	50	735	212	2.8	
	103	886	392	4.2	
	189	1096	540	4.5	
Nanostars	100	647,700,783	879 ^2^	10.7	[207]
Metamaterial	400 × 80 and 340 × 90 ^3^		588	3.8	[100]
***Silver nanoparticles***					
Nanosphere	40–90	400–480	160		[208]
Nanoprism	55–120	600–700	200–350	2.3–3.3	[208,209]
Nanoprism/Au coated	21–22	940	470		[210]
Array (NSL)	Varies	500–700	200		[211]
Nanocubes	30	430	1569 ^4^	5.4	[212]

^1^: Figure of Merit; ^2^: Value converted from eV RIU^−1^ to nm RIU^−1^; ^3^: H-fashioned cut-out structure in 30 nm gold film; ^4^: Value transformed from the unit of eV RIU^-1^ to nm RIU^−1^.

#### 3.2.2. Nano Plasmonic Molecular Rulers (PMRs) and PRET Biosensors

PMRs permit label-free estimation of different DNA and protein dimension and gap variations, instantaneous dynamic quantification of nucleic acid-protein ligand interactions, and confirmation of the existence of enzyme motion. PMR has a substantial advantage for durable kinetic studies in contrast to the Förster resonance energy transfer (FRET) method, as the conductive nanoparticles do not blink or photobleach [213]. Moreover, in contrast to FRET, which examines the binding events inside a 1–10 nm range, PMR deals with a separation space of up to 70 nm.

Ag or Au nanoparticle-based dimers have been employed for the measurement of DNA span together with hybridization kinetics. The space between two nanoparticles can be found based on the plasmonic coupling [214]. An additional study was dedicated to the study of nuclease activity, and wavelength shift was detected as a result of the variations in the dielectric constant with variations in DNA dimensions. In general, a 1.24 nm swing per base pair was detected [215]. Overlapping of the nanoparticle spectra and the absorption spectra of molecules supports the PRET, which results in spectral quenching as exposed in [216,217]. Additionally, a delicate and critical PRET basis sensor has been developed for Cu^2+^ ions [218].

#### 3.2.3. Nucleic Acid Hybridization Assays

**Figure 6:**
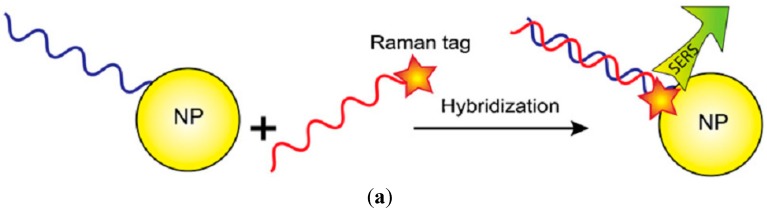

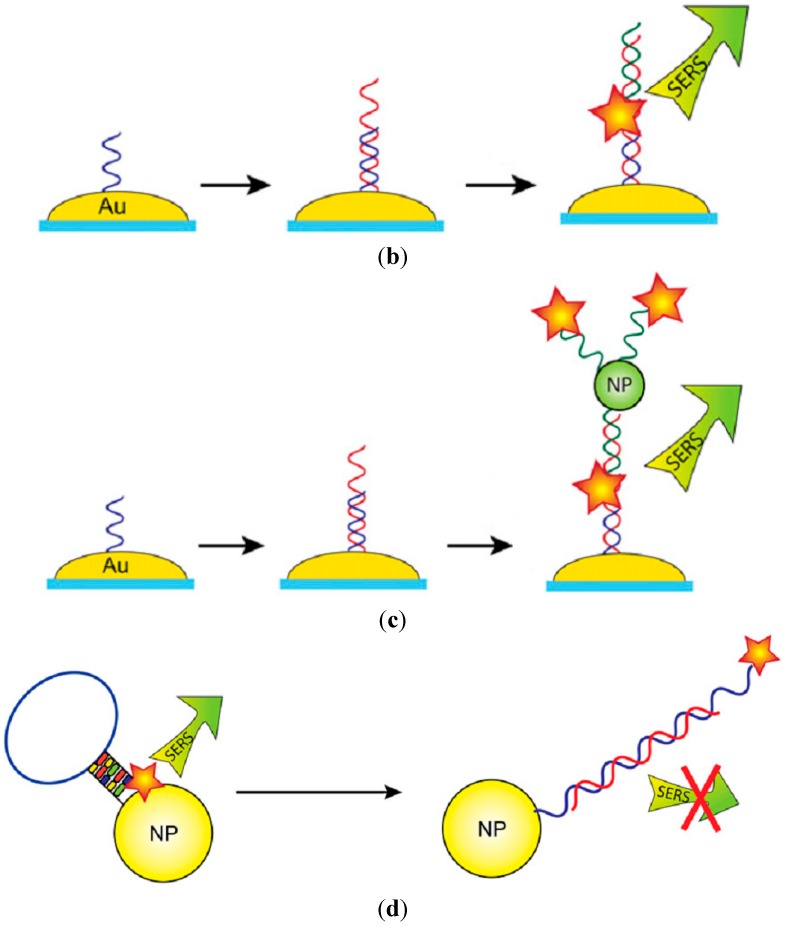
Different detection schemes using SERS. (**a**) DNA hybridization recognition by Raman tag; (**b**) direct recognition by Raman tag using nanoparticle array substrate for sandwich assay; (**c**) enhanced sensitivity by using secondary nanoparticle for sandwich assay; and (**d**) Molecular beacon for detection of a reduction in SERS signal.

Different bio-sensing and bioassay methods have been introduced by using nanoparticles for the recognition of the interactions of proteins, DNA hybridization, and different molecular actions. Huh *et al.* [219] identified DNA hybridization by using 50 nm Au nanoparticles in a microfluidic device that is operated by single stranded DNA (ssDNA). Here, passivation of nanoparticles was done using 6-mercapto-1-hexanol to reduce generic adsorption. Intended modifications of nucleic acids by hybridization and tetramethylrhodamine (TAMRA) brought the color into the near vicinity of nanoparticles (Figure 6a). In recent times, a sandwich assay using SERS for the purpose of DNA hybridization has been reported [220]. A capture ssDNA layer comprising thiols was restrained on a Ag nanoislands surface (Figure 6b,c). As a result of the use of nanoparticles for execution of the sandwich assay, the target recognition becomes enhanced from 1 nm to 0.4 fm.

Studies on molecular beacons were accomplished by modifying the DNA hairpin configuration through a Raman lively molecule [221,222,223]. This hybridization procedure interrupts the outline of the loop, which reduces SERS signal by growing the space of the Raman lively particle (Figure 6d).

#### 3.2.4. Protein Recognition Assays

The recognition and investigation of proteins is usually accomplished using nanoparticle combinations, Raman labels, and surfaces of the nanostructure [224]. Pavel *et al.* [225] have studied small proteins, which after contraction have two cysteine moieties at axially reverse locations, performing as ligands. With the existence of Ag nanoparticles, FynSH3 proteins prompted the gathering of aggregates and dimers, which consecutively delivered a boost for SERS. The connection points are called “hot spots” due to the inherently small (2.3 nm) protein size. This research demonstrated the implication of protein location regarding the region of “hot spot”. Formation of aggregates was also studied by Maher *et al.* [226] for different disease-specific enzyme recognition. Au nanoparticles were functionalized with N-fluorenyl-9-methoxycarbonyl (FMOC)-terminated peptide. The π–π connections among FMOC groups are the driving force of the aggregation and these could also be used as reporters with SERS. The prospective limit of detection for this scheme was likely as low as 10^−13^ M, while the trial data attained a LOD of 10^−11^ M, which is less than the essential range for biomedical uses. Au nanoparticles functionalized with thrombin were identified on heparin-adapted glass slides and anti-thrombin III [227], where a 10^−13^ M LOD was found at a SN^−1^ ratio of 3.

#### 3.2.5. Raman Labels

**Figure 7:**
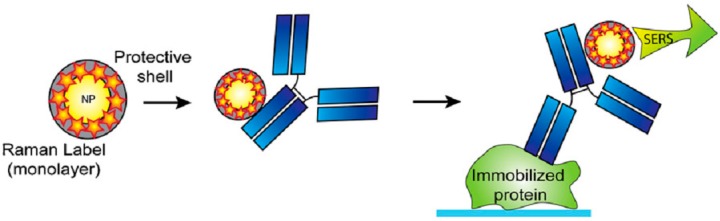
Graphical representation of immune-detection with Raman label encapsulation.

Different nanoparticles boost molecular Raman signatures while they themselves are in close vicinity. To increase the sensitivity, the coating of the huge amount of reporters has to use every nanoparticle. Gellner *et al.* [228] reported that a whole self-assembled monolayer (SAM) produces nearly 22 times stronger SERS in contrast to sub-monolayer coverage. SERS label confinement using organic polymers [229], proteins [230], and silica shella [231], increases solidity, removes the desorption probability of Raman reporters and reduces the generic adsorption. Encapsulated SERS assemblies, despite bigger dimensions, deliver a fabulous improvement in the intensities of SERS [232]. The conjugation of SERS labels with antibodies delivers recognition specificity and selectivity and is used in the immunoassays based on SERS [233,234]. A graphical demonstration of an immuno-histochemical assay using a SERS label is presented in Figure 7. Multiplexing was accomplished by these types of Raman labels with a dye range, and this technique is utilized for DNA recognition [232,233], and study of the interactions between different proteins and molecules.

#### 3.2.6. Intracellular Detection

Nanoplasmonic elements have been exploited for imaging cells, probing the interaction of drugs and cells, and photo-thermal treatment. Various nanostructures comprising Au nanospheres, nanoshells, and nanorods have been employed as thermal converters for initiating permanent impairment of different cancer cell lines [235].

Though biosensing inside cells with different LSPR biosensors is at the initial stages, these techniques are effective for diagnostics, drug distribution and checking the efficiency of therapies. Medication effects on the plasma membrane of active cells for antitumor drugs can be observed with a sensitivity of 10^−10^ M by SERS [236]. Kniepp *et al.* [237] studied the diffusion of drugs through the cell membrane with the use of Ag and Au nanoparticles that were supplied by endocytosis. Anticancer drug diffusion through cancerous cells was observed by Ag-layered silica beads in [238]. A SERS probes delivery method was revealed for the cell nucleus with a Raman reporter [239]. Breast cancer detection from blood samples has been done with a mixture of nanoparticles overcoated with epithelial and anti-her2 antibodies coupled with Au nanoparticles which possibly deliver SERS [240]. This technique delivered good sensitivity and high specificity using whole blood samples.

### 3.3. Challenges of Designing Optical Bio-Sensors

**Figure 8:**
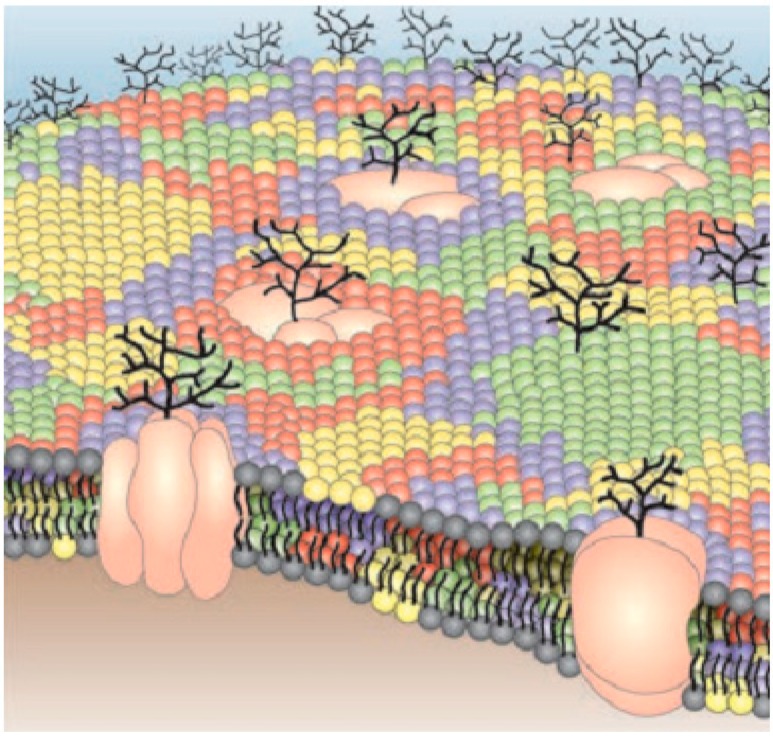
An illustration of a cell membrane in the nano-meter scale [243].

Although the future of optical biosensor technology depends on innovative researchers, the transition rate to the user community will be measured by various non-technical issues [241]. Moral apprehensions were also stated with respect to the genetic data usage and nanomaterial protection and such types of concerns about humans will eventually drive regulation. Different social considerations over problems, for instance depletion of resources will also force prime concerns for system strategy in addition to application ranges. The researchers who are working on the development of optical biosensors have a prime opportunity to integrate new knowledge into existing systems. The only restrictions appear to be the capability of integration of the elementary and innovative information with other disciplines, to gather skillful associates to support the effort, and to obtain economic and physical assets to investigate biosensors in the optical regime. Moreover, we need to study the critical users, consistency of the produced data, and the reaction to that data. We have listed below some points which we have to give emphasize before starting the design of optical biosensors:

(i) The ultimate challenges in designing biosensors are proper understanding about the correlation among the construction, operation and dynamics of different biomolecules in living cells. Though modern techniques have made massive improvements in detecting the components of different cells, detection of molecular procedures in living cells remains a main objective.
(ii) Different multi-molecular relations that command different cell functions happen at the nanometer scale [242,243] (Figure 8). This size regime is not reachable by classical optics with the diffraction of light, so we need a proper understanding of nanophotonics by which we can overcome the limitations of classical optics.
(iii) We need proper knowledge about the functionality and effects of nanoparticles before practical implementation.
(iv) Reproducibility and precise fabrication of resonant antenna‒without precise fabrication, it is not possible to achieve the desired sensitivity and accuracy.
(v) Once we have designed an optical antenna for biomedical applications, we must take time for enough study about the possible side effects. Therefore, a proper understanding about the effects of the specific antenna before practical implementation is a prerequisite [244].
(vi) Development of user-friendly experimental setups for widespread use of nanophotonics. France has identified some infrastructure constraints (Table 12) [241,245].

**Table 12:** Laboratory structure constraints and implication.

Constraints
Extensive difference of laboratory facilities among countries
Poor or missing peripheral quality control and laboratory certification systems
Erratic quality of reagents
Lack of important equipment
Shortage of consumables in laboratory
Undependable water supply and quality
Unreliable power supply and quality
Inconsistent capacity of refrigeration
Insufficient skilled staff
Limited training opportunities
Poor waste-management facilities

#### 3.3.1. Use of Nanoantenna Gratings for Tuning of Biosensors

The use of periodically corrugated metal-dielectric interfaces is a technique to overcome the wave-vector mismatch. The diffracted orders from the periodic corrugation have wave vectors larger in magnitude than those of the incident light. A light beam is directed towards a medium in which the surface has a spatial periodicity comparable to the wavelength of the incident light. The incident beam is diffracted, and the components of the diffracted light whose wave vectors coincide with the SP’s wave vector get coupled to the SP. Efficient coupling is provided to both air-metal and substrate-metal SP modes of a metal film, if the film thickness and the grating corrugation depth are properly allied.

The main benefit of grating-coupled biosensors is that they can be produced by mass replication technologies, such as injection molding and hot embossing. These technologies have have facilitated to produce low-cost and high-throughput biosensing platforms for label-free monitoring of biomolecular interactions [110,246].

#### 3.3.2. Human Exposure to Nanotoxicology

**Figure 9:**
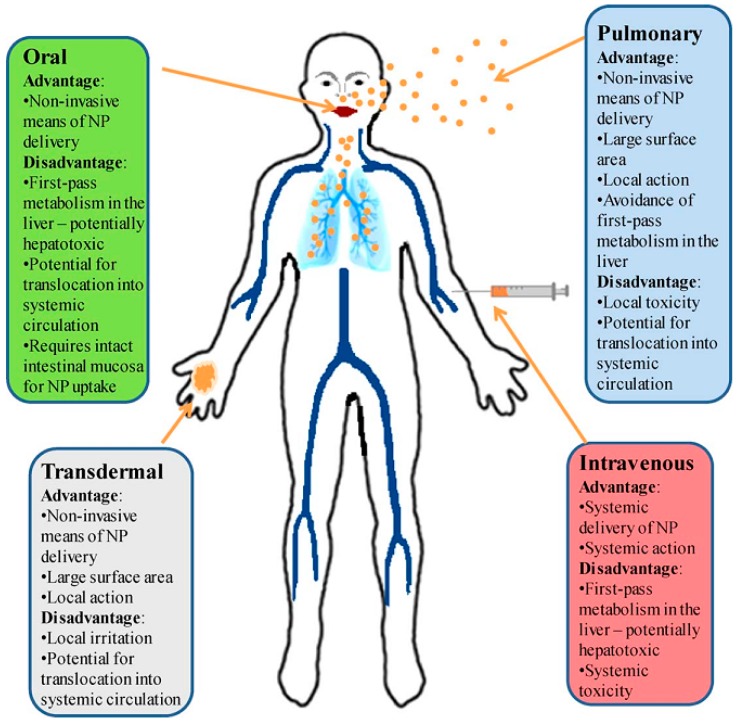
Nanoparticle administration paths and their advantages and disadvantages [247].

Conventionally, toxicology addresses hostile effects of poisoning due to chemicals on human beings, animals and the environment. Figure 9 represents the graphical view of the advantages and disadvantages of nanoparticles.

Many researchers have concentrated on developing nanoscale materials which might hold probable reasons for toxicity [247,248,249,250,251] and researchers must pay attention to this fact during the design and implementation:

(i) All of the nanostructures have their own optical, electronic, and magnetic properties associated with their dimensions. The breaking of these types of structures may cause a poisonous outcome.
(ii) The surfaces of the nanostructure take part in many oxidative and catalytic reactions. If these responses produce cytotoxicity, the deadliness becomes superior for greater surface-to-volume ratio. This surface-to-volume ratio increases when the diameter of a spherical particle becomes smaller and due to this increased surface area, the chemical reactivity is increased. This is most significant for nanobiological relations.
(iii) Many of the nanostructures have composites with well-known harmfulness and in consequence, the disintegration of these composites will possibly cause alike toxic reactions to the metals or composites themselves. Table 13 highlights the applications and concern surveys for the most frequently used nanostructures.
(iv) Traditionally, toxicology is related to the conception of dose and reaction to a dose. In nanotoxicology, the evaluation of appropriate and realistic dose ranges is essential for achieving significant results from public health risk assumption experiments. Therefore, the nano-toxicologist ought to test the toxicity of nanoparticles on the basis of practical doses in spite of the impractically high doses for achieving a biological reaction.
(v) The size of nanoparticles is at the same scale of protein molecules and they are capable of interfering with the signaling systems of cells. They also can interact with proteins, by chaperone-like action or by altering their configuration. This type of protein missformation leads to neurodegenerative infections. Inspecting probable missformation of proteins and macromolecules is vital for nanotoxicological research.

**Table 13:** Survey of dominant nanostructures, their applications and the biological areas of concern.

Nanostructures	Applications	Concerns	References
Metal nanoparticles	Contrast agents; drug delivery	Element specific toxicity; reactive oxygen species	[249,252]
Nanoshells	Hyperthermia therapy	None demonstrated	[252,253]
Fullerenes	Vaccine adjuncts; hyperthermia therapy	Antibody generation	[252,254]
Quantum dots	Fluorescent contrast agent	Metabolism	[255,256]
Polymer nanoparticles	Drug delivery; therapeutics	Unknown	[257]
Dendrimer	Guest supply of drug	Metabolic path	[252,258]
Liposomes	Drug supply; contrast agent vehicle	Hypersensitivity reactions	[252,257]

To avoid the abuse of nanoparticles in humans, it is necessary to carefully monitor the above described toxicity reasons through the whole process of biosensor design to implementation. Along with this, we have to use the toxicity and safety information of nanomaterials for risk evaluation and management. Outcomes from quick testing techniques must be made accessible for the assessment of the toxicity of nanoparticles to ease the nanoparticle risk assessment process. The conclusions of a cellular test scheme and prudently certified *in vitro* trials and high-throughput approaches using proteomics and genomics have to be made accessible for risk evaluation [259]. Some legal approaches of nanoparticle risk assessment are given below:

The environmental and health aspects of nanotechnology have only been concerns for the last decade or so since 2003, as there were no scientific peer-reviewed articles about such issues [260], and up to August 1, 2005, there were less than 10 papers on the safety evaluation of nanomaterials [261]. However, the article [254] attempted to trace the historical origin of toxicological research relating to nanoparticles and mentioned that the toxic effects of some nanoparticles were reported even two decades ago. 

One of the serious tensions before the introduction of nano-enabled products is that many stakeholders compare this with that of genetically modified foods, which faced unprecedented challenges in the recent past. A significant portion of the consumers refused to welcome these foods. Therefore, risk assessment processes for nanomaterials are important to convince the consumers about their safety. The risk and safety concerns of nanotechnology are almost contemporary with its emergence. The Organization for Economic Co-operation and Development (OECD) has identified seven types of risks associated with nanotechnology, *i.e.*: (a) business risks, due to marketing of products; (b) intellectual property protection risks; (c) political risks due to economic development of countries; (d) privacy risks due to unlimited use of sensors; (e) environmental risks due to nanoparticle release; (f) safety risks of workers and consumers; and (g) futuristic risks, e.g., human enhancement and self replicator [262].

The general practice is that both the hazard and exposure are to incorporate into a risk assessment paradigm, consisting of Hazard Identification, Hazard Characterization, Exposure Assessment and Risk Characterization in order to make appropriate risk management decisions [263] opined that the four steps of traditional chemical risk assessment procedure, *i.e.*: hazard identification, dose-response assessment, exposure assessment, and risk characterization may not be suitable to be used for assessment of nanomaterials. The Scientific Committee on Emerging and Newly Identified Health Risks of the European Commission (SCENIHR) suggested to improve the risk assessment process to address all hazards relating to the exposure of nanoparticles and for developing an improved system, there is no alternative to conducting more research. According to SCENIHR, such a system should target the routine determination of physical and chemical properties of nanoparticles, measure the representative exposure to free nanoparticles in the environment, *i.e.*, in air, soil and water, assess whether nanoparticles exacerbate pre-existing medical conditions and the movement of nanoparticles inside living systems.

The issue of risk assessment is crucial in assessing the health and environmental impacts of nanoparticles and the article [264] shared that the following things should be considered in risk assessment of engineered nanoparticles: “(a) exposure assessment of manufactured nanoparticles; (b) toxicology of manufactured nanoparticles; (c) ability to extrapolate manufactured nanoparticle toxicity using existing particle and fiber toxicological databases; (d) environmental and biological fate, transport, persistence, and transformation of manufactured nanoparticles; and (e) recyclability and overall sustainability of manufactured nanomaterials” [264].

In order to assess the possible risk out of nanoparticles, it is imperative to analyse the whole life cycle of the nanoparticles, which includes the understanding of the processes and materials used in their manufacture, the possible interactions between the product and the individual or the environment during the manufacturing stage or during the disposal stage. Simultaneously, this is also important to consider the defense system of the body of the persons who will be dealing with the nanoparticles (U.K. Nanotechnology Working Group, The Royal Society and the Royal Academy of Engineering. London, July 2004, at pp. 35–36).

In [259], the conclusion is that though there is no need to alter the current overall approach to the risk assessment of chemicals, the data gaps of engineered nanomaterials (ENM) risk assessment include: “(1) ENM aerosol standards and agreement on ENM key metrics; (2) dependable exposure scenarios, affordable monitoring technologies, exposure data and models; and (3) biomedical data on ENM translocation and toxicity, and associated testing strategies (which must be linked to the exposure scenarios)” [259].

The definition of nanomaterial seems to be crucial in the risk assessment process. The Joint Research Center of the European Commission assessed the working definitions available in different countries like the UK, USA, Canada, Australia, Denmark and in different organizations, like International Organization of Standardization, European Union, European Committee for Standardization and found that there are different scales used to define nanoscale and the nanoscale ranges from “1 nm to 100 nm”, “up to 200 nm (in two or more dimensions)”, “between 1 nm to 100 nm”, and “less than 100 nm” [265]. However, there are also counterarguments where it is claimed that for nanotechnology, the definition of “one size fit for all” may not be suitable and therefore, nanomaterials should be considered case by case [266].

European Food Safety Authorities are the first international body that included the determination of exposure and toxicity testing strategy. The Scientific Committee on Consumer Safety of the European Commission has released guidance for the assessment of nanomaterials in 2012.

In [267], the author developed a tool named “NanoRiskCat” to assist companies and regulators to assess hazards and exposure potential of consumer products containing engineered nanomaterials. This tool is quite helpful to assist stakeholders in making decisions about more information as to exposure and effect, which will further assist to decide on the safe use of nanomaterials.

An interesting development is that based on control banding, a system to assess risks in pharmaceutical industries, an online risk-banding tool, *i.e.*, Stoffenmanager Nano (version 1.0) was developed for the assistance of the employers and employees to prioritize health risks arising out of exposure to manufactured nano-objects (MNOs) [268].

In [269], the authors advocated for an integrated approach to specific risk analysis at work. They revealed some gaps in the whole process, *i.e.*, “restricted information, problems in relating nanotechnologies and production of nanomaterials to specific areas of application, efforts required to assess the hazards posed by nanomaterials in realistic exposure conditions, ethical issues about nanotechnology in the workplace expected to arise from today’s knowledge about the hazards of nanomaterials and the risks they may pose to workers” [269]. Therefore, “an integrated approach to research, cooperation, and communication strategies is indispensable, if we are to direct our efforts towards responsible and sustainable growth of nanotechnologies” [269].

Instead of setting the agenda to assess the risks of nanomaterials in general, a case by case approach is suggested in a number of researches. As a result, different countries have conduced risk assessments of different nanomaterials, especially those which are mostly used in consumer products and concluded that within the existing knowledge and research findings, such nanomaterials are not injurious to human health. For example, the National Institute of Advanced Industrial Science and Technology of Japan completed the risk assessment of titanium dioxide (TiO_2_), fullerene (C_60_) and carbon nanotubes (CNT). Similarly, the Australian National Industrial Chemicals Notification and Assessment Scheme (NICNAS) confirmed that TiO_2_ is not normally toxic.

## 4. Prospective and Conclusions

Optical antennas are the prevailing tool for the manipulation of light on a nanometer scale and they are also capable of delivering optimum control over transduction in the far-field region. Present optical antenna research is being motivated in particular by developments in nanofabrication technology and RF antenna analogies. Though various antenna conformations are currently being appreciated in the optical regime, it is going to be fascinating to observe how different antenna parameters, such as the impedance matching, are going to be redefined for different types of optical sources, like atoms and molecules. Optical antennas unite the quantum methods and photon sources by including fascinating new physics, for instance the breach of selection procedures and unconventional ways for robust pairing. The ideas of focused radiation and focused reception can be pragmatic to the photon emitters. Once the techniques of nanofabrication have been become mastered, a variety of applications will appear, including controlled single-photon sources for quantum information, light harvesting, energy conversion, efficient biosensors, data storage, nanoscale optical circuitry and optical imaging beyond 10 nm resolution.

This review has emphasized the principle and applications of optical biosensors. An appreciation of optical antenna basics offers chances of tuning and controlling the optical performance. Optical antennas have a robust reliance on the shape, size and composition of the nanoparticles which deliver an enhancement of biosensors’ sensitivity. A wide range of investigations on optical antenna are now dedicated to making substrates, which afford solid improvements of the EM field and deliver information about attaining control of optical properties by controlling the physical factors of nanoparticles. In spite of the different challenges for practical implementation of optical biosensors, the technical works show that importance in optical biosensor improvement continues to increase at a tremendous pace.
